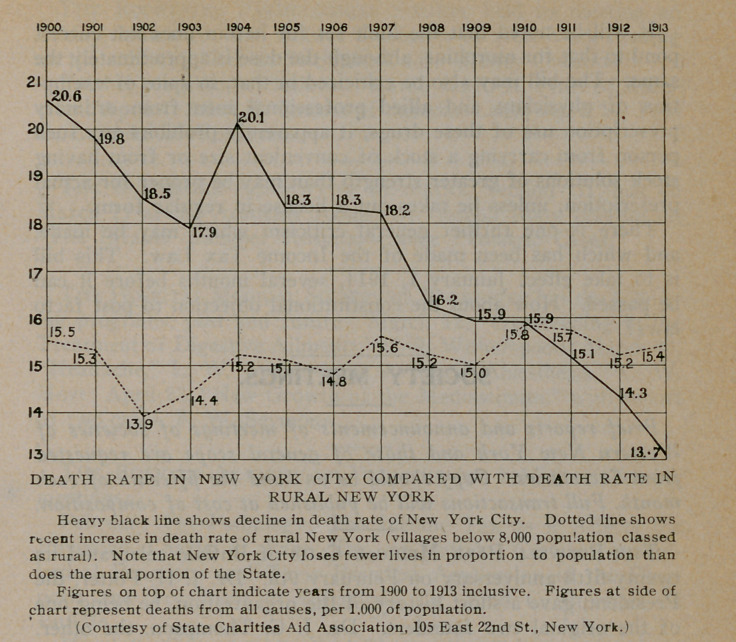# Topics of Public Interest

**Published:** 1914-04

**Authors:** 


### TOPICS OF PUBLIC INTEREST

### Infant Hygiene

According to the Seventh Annual Report of the New York Milk Committee, just issued· 41,000 baby lives have been saved in New York City by the systematic welfare work carried on during the last seven years by co-operating public and private agencies. During that time 950,000 babies have been born in New York City. If the infant death rate of the five years previous to the beginning of this work had prevailed there would have been 150,000 infant deaths instead of the 109,000 which actually occured. On the other hand, if New York's low death rate of 1913, i.e., 101.9 per 1,000 births, had prevailed throughout the seven-year period, only 96,000 babies would have died out of the 950,000 born.

The Committee points out that this record is not due to favorable weather conditions nor to other general accidental causes. Chicago, Philadelphia, Cleveland, St. Louis, Pittsburgh, Detroit, Buffalo, New Orleans and Toledo, all show an increase of deaths in 1913.

Another important step taken by the Committee, during the year, was the calling together of representatives appointed by the Governors of the eastern and middle states to discuss the question of uniform state regulation of the milk supply. Control bills were introduced in several states. The bill introduced in the New York State Legislature, while defeated by a small margin, aroused so much public sentiment that it will be reintroduced this winter, with strong prospects of success.

The Committee also held a conference to consider a method of fixing the market price of milk to the producer.

In systematic education of mothers in matters pertaining to feeding, hygiene and sanitation, the Committee's most important achievement during the year was the completion of its comprehensive experiment in the prenatal education of 3,300 mothers. In this experiment the stillbirths and the deaths under one month were reduced approximately twenty-five per cent. (25%). This work was carried on by visiting nurses in the homes of the expectant mothers.

The Committee also began a demonstration with a public Health Center which it believes will revolutionize methods of carrying on public health work in general, and infant welfare work in particular in large cities.

During the year the Milk Committee has given medical advice and attention at its centers to 8.088 mothers and babies. It has instructed mothers in their homes through 26,650 visits made by physicians and nurses. It has printed and distributed 300,000 educational circulars and 16,500 booklets and other educational matter.

---

New Buffalo Appointees. Dr. Andrew C. Callahan, City Physician, first district, $500, vice Dr. Harold J. McDonald, temporarily appointed Diagnostician. Also Dr. Charles S. Meahl, temporarily appointed Diagnostician, $2,000. John H. Stackhouse, D. D. S., temporarily appointed Dental Inspector, also Raymond J. Sandman, D. D. S., $500. Dr. Francis Argus, interne, Ernest Wende Hospital, $900. The temporary appointments have been made for newly created positions, in anticipation of civil service examinations.

The duties of the Diagnosticians are recited by the Health Board to be as follows:

To investigate all sudden deaths, not due to accident or violence, which are investigated by the county medical examiner, in accordance with the State law; to investigate all deaths where there is a question of diagnosis, cause unknown or where the cause of death is unsatisfactory, where no physician was in attendance or where the physician is out of town and no certificate can be obtained.

They shall also investigate all cases of skin diseases when requested to do so by any physician; they must investigate every case of chicken pox reported to the department of health; shall investigate all exposures to small pox; lift all small pox quarantines; enforce regulations governing small pox, and shall supervise all disinterments.

They shall also take cultures of people suffering from diphtheria who are unable or unwilling to have medical assistance at their own expense, who are not in the category of poor or needy and require the assistance of the city physician.

They will be on duty subject to call day and night and will be attached to the bureau of vital statistics.

---

### Automobile Notes

Capacity of Horizontal Cylindric Tank. The general problem is to determine the area of segments of a circle, the whole circle being multiplied by the long axis of the tank to find the volume of the cylinder. If the total contents of the tank are known, as is usually the case, the circle may be assigned a value corresponding to the volume of the cylinder. The known quantity is the depth of gasoline, as determined by sounding. This is the versed sine of half the arc subtended. The semi-diameter of the circle, minus this depth is the cosine, from which the arc may be obtained by a table of natural or logarythmic sines and cosines. The segment consists of the corresponding sector minus the empty triangle above. The sector is a fraction of the whole circle, corresponding to 360 degrees divided by the number of degrces in the arc subtended (double the arc or angle obtained from the versed sine). The empty triangle is the product of the sine and cosine of the half-arc. As the former is a relative quantity, the latter an absolute, they must be reduced to the same basis for subtraction, remembering that the total value of the circle is 3.1416 times the square of the radius.

By slide rule, the following nearly exact values have been found for ten-gallon tanks, with a ten-inch diameter, by half inches of depth of gasoline. The formulæ may be generalized by reading inches as tenths of any diameter and amounts of gasoline as parts of the total capacity, moving the decimal point one place to the left. For example: 3.5 inches, 3.127 gallons becomes 0.35 (35/100) of diameter corresponds to 0.3127 of the total capacity.

| | | | | | |
|---|---|---|---|---|---|
| 0.5 | inch...... 0.173 | gallons | 5.5 | inches...... 5.686 | gallons |
| 1. | inch...... 0.48 | gallons | 6. | inches...... 6.265 | gallons |
| 1.5 | inch...... 0.96 | gallons | 6.5 | inches...... 6.873 | gallons |
| 2. | inch...... 1.43 | gallons | 7. | inches...... 7.425 | gallons |
| 2.5 | inch...... 1.958 | gallons | 7.5 | inches...... 8.042 | gallons |
| 3. | inch...... 2.575 | gallons | 8. | inches...... 8.57 | gallons |
| 3.5 | inch...... 3.127 | gallons | 8.5 | inches...... 9.04 | gallons |
| 4. | inch...... 3.735 | gallons | 9. | inches...... 9.827 | gallons |
| 4.5 | inch...... 4.314 | gallons | 10. | inches......10. | gallons |
| 5. | inch...... 5. | gallons | | | |

The following proportions, worked out in a slightly different way may also be of interest:

| | | |
|---|---|---|
| Exactly 1/4 of diameter................ | approx. 1/5 | of bulk |
| Exactly 1/2 of diameter................ | exactly 1/2 | of bulk |
| Exactly 3/4 of diameter................ | approx. 4/5 | of bulk |
| Approx. 1/7 of diameter................ | approx. 1/10 | of bulk |
| Approx. 6/7 of diameter................ | approx. 9/10 | of bulk |
| Approx. 3/8 of diameter................ | approx. 1/3 | of bulk |
| Approx. 5/8 of diameter................ | approx. 2/3 | of bulk |

VITAL STATISTICS OF NEW YORK STATE, WITH SPECIAL REFERENCE TO RURAL MORTALITY. The New York State Department of Health gives the following statistics.

| | Death rate per 1000 population 1912 | 1913 |
|---|---|---|
| Greater New York | 14.1 | 13.7 |
| Rest of State | 15.5 | 15.7 |
| Death rate of cities outside of Greater New York | 15.7 | 16.1 |
| Rural death rate* | 15.3 | 15.4 |
| Whole State | 14.8 | 14.7 |

*Places under 8,000 population.

Total deaths and death rates from principal causes were as follows:

| Cause of death | Total deaths | Death rate per 100,000 population | Percent-of all deaths |
|---|---|---|---|
| Pulmonary tuberculosis | 13,811 | 139.7 | 9.5 |
| | (13,716) | (142.9) | (9.6) |
| Bright's disease | 10,430 | 105.4 | 7.1 |
| | (10,613) | (110.5) | (7.5) |
| Lobar pneumonia | 9,302 | 94.1 | 6.4 |
| | (9,560) | (99.5) | (6.7) |
| Accidents | 8,512 | 86.1 | 5.9 |
| | (8,130) | (84.6) | (5.7) |
| Cancer | 8,528 | 86.2 | 5.9 |
| | (8,250) | (85.9) | (5.8) |
| Diphtheria | 1,854 | 18.7 | 1.27 |
| | (1,624) | (16.9) | (1.1) |
| Old age | 1,686 | 17.0 | 1.16 |
| | (1,795) | (18.7) | (1.3) |
| Typhoid fever | 1,018 | 10.3 | 0.7 |
| | (1,128) | (11.8) | (0.8) |
| Scarlet fever | 837 | 8.5 | 0.57 |
| | (789) | (8.2) | (0.55) |
| Measles | 1·071 | 10.8 | 0.73 |
| | (1,050) | (10.9) | (0.74) |
| Whooping cough | 818 | 8.3 | 0.56 |
| | (683) | (7.1) | (0.48) |
| Erysipelas | 497 | 5.0 | 0.34 |
| | (532) | (5.6) | (0.37) |
| Meningitis—epidemic | 312 | 3.2 | 0.22 |
| | (333) | (3.5) | (0.23) |

Figures in parenthesis are for 1912.

Thirteen diseases—40.35 per cent. of all deaths.

We have been much impressed with the fact that, in recent years, the mortality of cities has diminished so as to show a lower death rate than the country. This is a logical result of co-operative sanitary measures in cities and the persistence of lack of plumbing, facilities for drainage from privies and barnyards, pig styes, etc., into wells, exposure to cold and damp and to accidents, poor diet, etc., in the country. But, as we pointed out last year· the highest death and disease incidence are usually found in the small towns and villages. It occurred to us to calculate the death rates for "the rest of the county," after explicit mention of cities and certain villages in the health reports and the result has been somewhat surprising. It should be remembered that the rural districts in census and these health reports include villages of less than 8,000 population, and it is scarcely necessary to point out that, either in the social or by the hygienic sense, a village of more than a few hundred is a very different institution from a farming community. Even "the rest of the county" does not consist of open country, but it comes nearer the true conception of a rural district than the definition adopted in vital statistics.

| | | | |
|---|---|---|---|
| Suffolk | 23 99 | Ulster | 13.72 |
| Westchester | 17.19 | Clinton | 13.32 |
| Albany | 13.72 | Essex | 16.59 |
| Columbia | 14 07 | Franklin | 14.61 |
| Dutchess | 19.68 | Erie | 12.82 |
| Green | 15.21 | Monroe | 13.60 |
| Orange | 11.42 | Niagara | 12.51 |
| Putnam | 12.00 | Orleans | 12.89 |
| Rensellaer | 15.43 | Oswego | 17.35 |
| Rockland | 11.39 | Wayne | 13.17 |

Even with a table of logarythms, such calculations are tedious, and, as we may recall from our school days, New York has an unreasonably large number of counties. We have not taken the trouble to make the full computations, but have taken the first and last parts of the State list as they run. This ought to make a fair basis for comparison. Suffolk, Westchester, Dutchess, Essex and Oswego counties show high death rates, for which we are inclined to believe local factors, probably involving village sanitation and the inevitably high mortality of asylums of various kinds, may be adduced as explanation. For the typic rural parts of counties, even without being able to exclude villages, the mortality is decidedly low. Facts are better than theories. We trust that, since the hygienic and sanitary factors of the real country, the small town and the large city involve distinct differences, future statistics may show the conditions as they actually exist.

TWELFTH INTERNATIONAL OPHTHALMIC CONGRESS AND ANTI-SEMITISM.—Prof. Julius Hirschberg, President of the Berlin Ophthalmologic Society, is in correspondence with various constituent societies of the congress, in an endeavor to induce both Jews and Gentiles to signify their intention not to attend the Congress.

"The ministry of the interior has given consent to the free admittance of the Jewish members of the congress, but prescribes that there shall be put upon the pass of each such member by the central bureau in St. Petersburg a notation with regard to the length of time the holder is permitted to live within the boundaries of the empire. The last date for the sojourn of such members in Russia will probably have to be September 15, with which date, moreover, the excursion rates for the trip to the congress also end."

We do not blame the Jewish members of the Congress for feeling offended at the Russian attitude, and it might, perhaps, lead to a revision of Russian sentiment and even law, if an influential body of scientific men, of all nations and various races and religions, took this method of reminding Russia that it has not caught the spirit of liberality of modern civilization.

However, as a matter of fairness, there is something to be said on the other side. The Jews should remember that European—including American—civilization is only about 1500 years removed from bronze age savagery, whereas they themselves are at least four times and possibly ten times as far away from this stage of development. Without substituting one form of race prejudice for another, the fact remains that different races have progressed at very different rates. For example, some of the Australian aborigines are said to be still in the palæolithic stone age. In the last twenty or thirty centuries, the Aryan races have, on the whole, not only distanced all competitors, but have passed, quite rapidly, many races originally far ahead of them. Not all Aryan races have made equal progress even with the mutual assistance of the last few centuries. Whether the Russians are essentially Slavs or not and whether the Slavs are a branch of the Aryan stock or not, the fact remains that Russia is, in many respects, two or three centuries behind the average European nation in conceptions of social government. From its own standpoint, Russia has made a concession to the Jewish members of the Congress, and while this concession is itself an insult from the standpoint of the 20th century in America and western Europe, it does not involve any practical hardship, and it is probably a favor extended in good faith from the point of view of the average Russian. It seems to us an open question whether the Congress should be broken up as a protest against an inevitable unevenness of world progress or whether a wiser policy would be for both Jews and Gentiles to attend, thus affording a timely, present object lesson in the good work accomplished by the former race and of the mutual esteem and harmony engendered by co-operation without regard to race.

---

Comparative Danger of Vehicles. Chicago statistics, while confirming the popular opinion that more accidents have been due to automobiles than to horse-drawn vehicles, of late years, show that the street car, in spite of its being confined to a known track, is still more deadly, and that in relation to efficiency, in the last four years, up to January 1, 1914, motor vehicles have caused the lowest amount of accident and death.

| | No. | Av. daily mileage. | T'l daily mileage. |
|---|---|---|---|
| Horse vehicles .......... | 65,118 | 12 miles | 781,416 |
| Power vehicles ......... | 37,406 | 42 miles | 1,571,052 |

Average of accidents in four years:

| | T'l accid's 1910 to 1914. | Accid's per day | Ave. accid's per 5,000,000 miles. |
|---|---|---|---|
| Horse vehicles ......... | 6,047 | 4.15 | 26.55 |
| Power vehicles ......... | 5,784 | 3.96 | 12.6 |

Accidents reported to police department:

| | Street railway. | Horse vehicle. | Power vehicle. | Total per year |
|---|---|---|---|---|
| 1910 ............... | 3,969 | 1,596 | 998 | 6,563 |
| 1911 ............... | 3,664 | 1,561 | 1,153 | 6,378 |
| 1912 ............... | 4,106 | 1,507 | 1,604 | 7,217 |
| 1913 ............... | 4,283 | 1,383 | 2,029 | 7,695 |
| Totals ............ | 16,022 | 6,047 | 5,784 | |

Coroner's cases or fatalities:

| | Street railway. | Horse vehicle. | Power vehicle. | Total per year |
|---|---|---|---|---|
| 1910 .................. | 175 | 67 | 52 | 294 |
| 1911 .................. | 161 | 75 | 75 | 311 |
| 1912 .................. | 209 | 49 | 96 | 356 |
| 1913 .................. | 165 | 44 | 136 | 345 |
| Totals.............. | 710 | 235 | 361 | |

AUTOMOBILE CENSUS. It is estimated that, in 1913, there were 1,128,000 automobiles owned in the continental United States. The population, at 2 per cent. increase per annum since 1910, would be about 97,400,000. The ratio is one automobile to 86 persons, or one to about 13 average families. There were more than twice as many automobiles as there were incomes reported beyond the exemption limit. Of course, many rich families own several and many automobiles are commercial, but the fact remains that a great many persons keep automobiles who cannot afford them, and this undoubtedly has an influence on the cost of living and the general complaint of hard times.

---

HONEST GASOLINE. Alderman Burley of Buffalo has introduced a resolution for the preparation of an ordinance requiring dealers in gasoline to state its test and to subject them to inspection by the city inspector of oils, and to a penalty for misrepresentation.

---

SMALL POX. On March 2, a Lehigh Pullman porter was found to be infected with small pox. He was sent to the isolation hospital and the car was disinfected.

---

NEW YORK SKIN AND CANCER HOSPITAL, Second avenue, corner 19th street. Clinical lectures at 4.15 o'clock. Syphilis, by Dr. Bulkley. April 1, Primary Lesions, Genital and Extra-genital—Innocent Syphilis; April 8, Early Manifestations of Syphilis; April 15, Late Manifestations of Syphilis; April 22, Marital and Hereditary Syphilis; April 29, Treatment of Syphilis. Cancer, by Dr. William Seaman Bainbridge. April 30, Some Practical Phases of the Cancer Problem. The lectures will be illustrated by cases, models, colored plates, photographs, etc. The course will be free to the Medical profession, on the presentation of their professional cards.

---

CONVICTION OF ABORTION. Dr. Alice Gertrude Sharon of Batavia has been sentenced to 1-1½ years in Auburn prison, on account of the death of a patient on September 25. She declared her innocence at the time of the sentence.

---

TYPHOID EPIDEMIC. St. John's, Iberville and Sabrevois counties, Quebec, have an epidemic of rather mild typhoid, though several deaths have occurred. Of a total population of 7,000, over 2,000 have been infected. Many of the Royal Canadian Dragoons have contracted the disease. We hope to have a more detailed account from Associate Editor Dr. J. George Adami in the next issue.

Tame Physicians In Drug Stores. The New York County Medical Society has won a preliminary victory in a crusade against drug store practice of medicine, even when the store employed a physician. Such a physician, arrested as violating the present State law, obtained a writ of habeas corpus, which was dismissed on argument in the Supreme Court. The druggists, however, expect to carry the contention to the Court of Appeals. While our sympathies are strongly in favor of medical ethics, it occurs to us that the druggists may eventually win this fight, as, otherwise, the law would be assumed to dictate the location of physicians' offices in general and the terms on which they carried on business.

---

Permanent Home For College of Surgeons. The American College of Surgeons has already taken up this matter. New York, Boston, Cleveland, Washington and Minneapolis are mentioned as sites, Chicago to be avoided because the headquarters of the A. M. A. are there.

---

Cancer In X-Ray Tube Workers. In 1906, John L. Bauer, a glassblower employed by Henry Green, a pioneer X-ray tube manufacturer of Hartford, died of cancer. On March 4, 1914, Mr. Green died of cancer of the liver. Cancer apparently due to exposure to X-rays and radium emanations, etc., is fairly frequent. However, 6-9 per cent. of all deaths at ages over 45, occur from cancer, so that it is wise to study statistics carefully before reaching a conclusion. And it is equally wise to be careful in the use of these emanations till the statistics are accurately compiled.

---

Tuberculosis Hospital For Children. $125,000 bond issue has been approved by the Buffalo Councilmen for erecting a special children's hospital in connection with the J. N. Adam Memorial Hospital at Perrysburg. The new building is to accommodate 100 cases, equally divided between pulmonary and glandular.

---

Sex Hygiene. Supt. Henry P. Emerson of Buffalo has declared against teaching sex hygiene in the public schools.

---

Tape Worm as an Obesity Cure. The *Journal of the N. A. R. D.* prints a story to the effect that tænia larvæ have been found in certain high-priced obesity capsules—a case of the cure being worse than the disease.

New Traffic Laws. After considerable opposition, Buffalo has prepared an ordinance requiring all vehicles to carry a light after dark. A similar law applies to the State, except that vehicles used mainly for carrying hay and straw are exempt. In Buffalo, searchlights must not exceed twenty candle power. All vehicles must stop for passengers boarding or leaving street cars unless there is a ten-foot clearance from the platform. Material, such as pipes and lumber, projecting beyond a wagon box, must be marked by a red flag by day, by a red light at night. Automobiles may stand for an hour on down-town streets and for an indefinite period elsewhere. All of these points have been specifically advocated in this Journal as matters of life-saving importance.

We venture to make one suggestion further: If we were a pedestrian on an automobile route at night, we would walk on the left side of the road, so as to be able to see an approaching vehicle. The special reason is this: An automobilist, passing another automobile which carries bright head-lights, cannot see a dark object in front of him.

---

Ratio of Medical Staff to Personnel in Army and Navy. The U. S. Army has a total of about 85,000, with about 600 surgeons; the Navy, 51,000 with 325 surgeons—the respective ratios being about 1:140 and 1:160. In civil life the ratio, allowing only for physicians actually in practice, is about 1:700. It should be remembered that the army and navy must provide for sudden demands of warfare and epidemics and that the duties of medical officers include much hygienic instruction, executive work, and, for a considerable number, more or less special details for teaching and receiving instruction, research work, etc. Approximately one per cent. of the entire medical profession of the country is employed in the army, navy, marine corps and public health service. About the same proportion is employed in various civil service positions. It is probably a fair estimate that 5 per cent. of the entire profession is at present employed on contract by various branches of the National, State and local government.

---

Surgeon General of the Navy. Medical Inspector Wm. C. Braisted has been promoted to succeed Charles S. Stokes, retired. Dr. Braisted was born in Ohio, October 9, 1864, and was appointed to the Navy in 1890.

---

Index Office. Under this title, a bureau has been organized in Chicago to prepare bibliographies, translations, abstracts, etc., to secure illustrations for literary work and to bring investigators in touch with others interested along the same lines. Address Aksel G. S. Josephson, Secretary, 31 W. Lake St., Chicago.

The Buffalo Chamber of Commerce, through Mr. Henry B. Saunders, Commissioner of the Convention Bureau, thus cordially supports the invitation of the local profession to the State Society:

"Buffalo leads the world in its equipment for the proper care of conventions, and no other city combines so many conveniences and attractions. Buffalo is the only city which has two fine halls municipally owned and open for worth-while conventions, and they have become an invaluable asset in bringing large gatherings here, so that Buffalo, in the past three years, has advanced into the front rank of convention cities, entertaining more national and international state and interstate gatherings than any except a few of the larger cities.

At the annual meeting of the Medical Society of the State of New York, in New York City, April 28-30, invitations will be extended by the city, through Mayor Fuhrmann, the Chamber of Commerce and the Medical Society of Erie County, asking the State Association to come to Buffalo for its annual meeting in 1915, and it is expected that the invitation will be accepted.

It has occurred to several local members that because of our unusual facilities the Buffalo convention of 1915 might be made the occasion of a great commercial exhibit of surpassing interest to the members of the profession and valuable to manufacturers and dealers. Either of our two splendid convention halls would be available for this purpose. Elmwood Music Hall would afford an ideal setting for such an exhibition, but it might prove that the Broadway Hall would be needed, because it has much more space available. The Broadway Hall was rebuilt especially for exhibition purposes and is the best equipped show building in the United States. It is exceptionally well lighted by day or night, and conduits have been laid under the floor, with outlets on 25-foot centers, so that electric power is available under almost any arrangement of booths, doing away with dangerous and unsightly overhead installations.

It might appear that the 50,000 square feet of space on the main floor of this building would be too large for the proposed exhibition, but this is not the experience of other organizations which have used the hall, its conveniences appealing very strongly to exhibitors.

Only this year, when the Broadway Hall was offered to the Manufacturers' Auxiliary of the Fraternity of Operative Millers, they thought the space too large, because it is much greater than they had used in the past. However, announcements were sent out late in January, outlining the possibilities of display in the hall, and by March 15 all the space had been sold and it became a problem of allotment. This exhibit does not take place until the first week in June.

Broadway Hall is conveniently located and is within a few minutes walk of Main street and the principal hotels, and it would seem that it affords the opportunity for the greatest and most interesting convention ever held by the State Association.

Little need be said of our other facilities for a successful convention. Buffalo has ample hotel accommodations, and her best hotels are not excelled in appointments or service by those of any other city. Social service here is on a high plane, and we have, for the relief of the sick, twenty public hospitals, many of them models of their kind, together with six dispensaries, an efficient district nursing association and visiting tuberculosis nurses.

Buffalo is rich in historic associations, and her splendid public buildings, parks, drives and streets are a delight to many thousands of visitors every year."

---

Medical Bills Before the N. Y. State Legislature. The Committee on Legislation of the State Society calls attention to the following:

Senate Bill No. 325 by Mr. Boylan, in relation to habit forming drugs—Opposed.

Assembly Bill No. 465 by Mr. Conkling and Senate Bills Nos. 608 and 631 by Mr. Herrick, amending the Medical Practice Act and licensing Osteopaths to practice medicine (reported to Committee of the Whole)—Opposed.

Senate Bill No. 281 by Mr. McClelland and Assembly Bill No. 1148 by Mr. Jones, to license Naturopaths to practice—Opposed.

Senate Bill No. 710 by Mr. Boylan and Assembly Bill No. 963 by Mr. Kerrigan, known as the Chiropractic Act—Opposed.

Take Notice—The aim of these bills is to destroy the present Medical Practice Act and permit persons not properly qualified to practice medicine.

Senate Bills No. 3 by Mr. Boylan and No. 182 by Mr. Herrick and Assembly Bill No. 501 by Mr. Gallup, Antivivisection bill, with the aim to abolish vivisection—Opposed.

Senate Bill No. 575 introduced by Senator Seeley, to amend the Public Health Law, relative to medical licenses—Opposed, unless the bill is amended as follows:

Omit the power of the Regents to decide what is ethical;

Omit giving the Regents power to reinstate a physician convicted of a felony, if his conviction has been for conduct in his professional capacity.

Kindly *favor* the passage of Assembly Bill No. 905 by Mr. Brennan, abolishing the office of Coroner and creating the offices of Medical Examiners, etc., and Senate Bills Nos. 208 and 209, introduced by Senator Seeley (M. D.,) Craig Colony Bills, to improve present conditions.

Trap For Fly Larvae. The U. S. Bulletin of Agriculture, No. 14, February 28, 1914, by Robert H. Hutchison, suggests a method depending on the habit of the larvæ to migrate to moist portions of manure. The manure is stored over a coarse sieve or in a wire basket, sprinkled daily, and the larvæ are drowned in a container of water below. By covering the mass with dark cloth, flies were directed toward traps as they hatched and many of the mature flies were caught also. Nearly 99 per cent. of fly larvæ were collected within a week, in experiments, the total being estimated by adding the number of mature flies also caught. Of course, various practical points, as the depth of manure to which the method may be applied successfully, the time which it should be kept, inforcement of regulations, etc., remain to be determined.

---

Physicians' Bills For Work During Small Pox Epidemic at Niagara Falls. Dr. Edward Clark of Buffalo was paid $500 for twenty days' expert services, and various local physicians received compensation at the rate of $15 a day for house to house canvasses. The bills rendered were for approximately double these amounts.

---

Nationality of Civil War Soldiers. It is estimated approximately that about one-third of the present population of the U. S. is American in the colonial sense. As there was relatively little immigration for a period of about a hundred years from 1720 to 1820, the division is more logical than might seem. Even at the time of the Civil War, there was not only a considerable foreign population, but a good many of the first generation born here and a very marked development of loyalty to the adopted country, as the following statistics compiled by Benjamin Apthorpe Gould in 1868, shows:

| | |
|---|---|
| Native Americans | 1,523,267 |
| German born | 176,817 |
| Irish born | 144,221 |
| British Provinces in North America | 53,532 |
| English born | 45,508 |
| Other foreign born | 74,855 |
| | 2,018,200 |

---

United Twins Separated. The French twins, Madeleine-Suzanne, born November 28, 1913, were separated March 5, 1914, by Dr. Doyen of Paris. In 1902, he separated the Hindoo twins, Radica-Roodica, who died later of tuberculosis.

THE HARRISON ANTI-NARCOTIC BILL, now before the U. S. Senate, provides for the registration, at a fee of $1.00 per annum, of every person who produces, imports, manufactures, compounds, deals in, dispenses, sells, distributes or gives away, opium, or coca leaves or any compound . . . . or derivative thereof. A maximum, for each prescription for internal use of two grains of opium, ¼ grain of morphine, 1/12 grain of heroine, 1 grain of codeine per fluid or avoirdupois ounce is exempt. Strictly external preparations of opiates are exempt from these limits, but the same limits seem to apply to any preparation of cocaine or artificial substitutes, whether for internal or external use. Heavy penalties are provided for violation or evasion of the law.

The Act does not apply to physicians, dentists, veterinary surgeons employed to prescribe for particular patients receiving such drug or article receiving in good faith such drug or article. Nor does it apply to a pharmacist, who receives a prescription from a physician, dentist or veterinary surgeon, but pharmacists are required to preserve the prescriptions for a period of two years.

It will be noted that the limit set for heroin does not correspond to that for morphine, although the dose is approximately the same. The bill may also be criticized in that, in spite of exemption of physicians and allied professional men from ordinary prescription use of these drugs, it apparently prohibits any such person from carrying a stock of convenient size or from having stock solutions of greater strength than may be proper for actual prescription, unless he takes out a license in regular form.

There is one further general criticism which may be made, and which has been made of the Income Tax Law. This bill is to take effect January 1, 1914, several months before it can be passed. How about the constitutional objection to post facto laws?

## Figures and Tables

**Figure f1:**